# Successful Development of Small Diameter Tissue-Engineering Vascular Vessels by Our Novel Integrally Designed Pulsatile Perfusion-Based Bioreactor

**DOI:** 10.1371/journal.pone.0042569

**Published:** 2012-08-03

**Authors:** Lei Song, Qiang Zhou, Ping Duan, Ping Guo, Dianwei Li, Yuan Xu, Songtao Li, Fei Luo, Zehua Zhang

**Affiliations:** Department of Orthopaedics, First Affiliated Hospital, Third Military Medical University, Chongqing, People’s Republic of China; Okayama University, Japan

## Abstract

Small-diameter (<4 mm) vascular constructs are urgently needed for patients requiring replacement of their peripheral vessels. However, successful development of constructs remains a significant challenge. In this study, we successfully developed small-diameter vascular constructs with high patency using our integrally designed computer-controlled bioreactor system. This computer-controlled bioreactor system can confer physiological mechanical stimuli and fluid flow similar to physiological stimuli to the cultured grafts. The medium circulating system optimizes the culture conditions by maintaining fixed concentration of O_2_ and CO_2_ in the medium flow and constant delivery of nutrients and waste metabolites, as well as eliminates the complicated replacement of culture medium in traditional vascular tissue engineering. Biochemical and mechanical assay of newly developed grafts confirm the feasibility of the bioreactor system for small-diameter vascular engineering. Furthermore, the computer-controlled bioreactor is superior for cultured cell proliferation compared with the traditional non-computer-controlled bioreactor. Specifically, our novel bioreactor system may be a potential alternative for tissue engineering of large-scale small-diameter vascular vessels for clinical use.

## Introduction

Peripheral vascular disease becomes an increasing health and socio-economic burden in most countries. Surgical bypass with autologous vessels remains the main treatment nowadays. However, usable vessels are not often available due to vascular disease, amputation, as well as previous harvest [1]. Thus, small-diameter (<4 mm) vascular substitutes are urgently needed for patients requiring replacement of their peripheral vessels.

The most common alternative to autologous grafts is the use of synthetic small-diameter vascular grafts made of materials such as Dacron or expanded polytetrafluoroethylene [2]. However, the small-diameter arterials of low flow pose a different set of design criteria and introduce various problems including thrombogenicity not encountered in large-caliber vascular substitutes where these synthetic vascular grafts have succeed [3]. As a result, tissue engineering emerges as a promising approach for development of small-diameter vascular grafts. The main objective is to generate biological substitutes of small-diameter arterial conduits with functional characteristics of native vessels with cellular components.

Many challenges exist in the tissue engineering of small-diameter vascular grafts. The greatest one is the development of functional grafts with high patency as well as the antithrombotic properties [4]. Furthermore, it will be meeting all the criteria for tissue engineering of functional large-diameter vascular grafts which should possess mechanical properties such as high burst strength and simultaneous high compliance. In addition, there still exists a barrier to widespread application and low-cost mass production.

Current strategies for vascular tissue engineering use arterial wall cells including endothelial cells (ECs) and smooth muscle cells (SMCs) with or without a biodegradable scaffold. Bioreactors technology and bioprocess engineering principles, which can impart physiologically similar biochemical and mechanical stimuli to engineered grafts, are well accepted to facilitate the aseptic growth and maturation of functional grafts [5]. These physiologically similar stimuli are necessary for development of functional vascular grafts, as ECs and SMCs are significantly influenced by local fluid dynamics. Both shear stress and stretch stress are vital to growth and maturation of ECs and SMCs [6], [7]. Perfusion-based culture bioreactors have many advantages in vascular tissue engineering, as complex chemical and mechanical stimuli essential to appropriate development can be better achieved in a controllable manner than traditional static cell culture systems [8]. Many perfusion-based devices have been developed in which simple mechanical stimuli could be applied simultaneously, however, these systems provide only a limited ranges of mechanical conditions. Specifically, though previous studies have made great efforts in development of bioreactors to produce small-diameter vascular grafts, there still have not been adequate data of functional vascular characters to confirm the feasibility of these bioreactor systems.

In the present study, we successfully develop an integrally designed vascular bioreactor system. With the computer-controlled manipulation, this system allows precise adjustment of physiological pressure, and is capable of developing functional small-diameter vascular vessels with high patency as well as antithrombotic properties. Besides, this integrally designed computer-controlled bioreactor system may make industrial large-scale production of functional small-diameter grafts come true.

**Figure 1:**
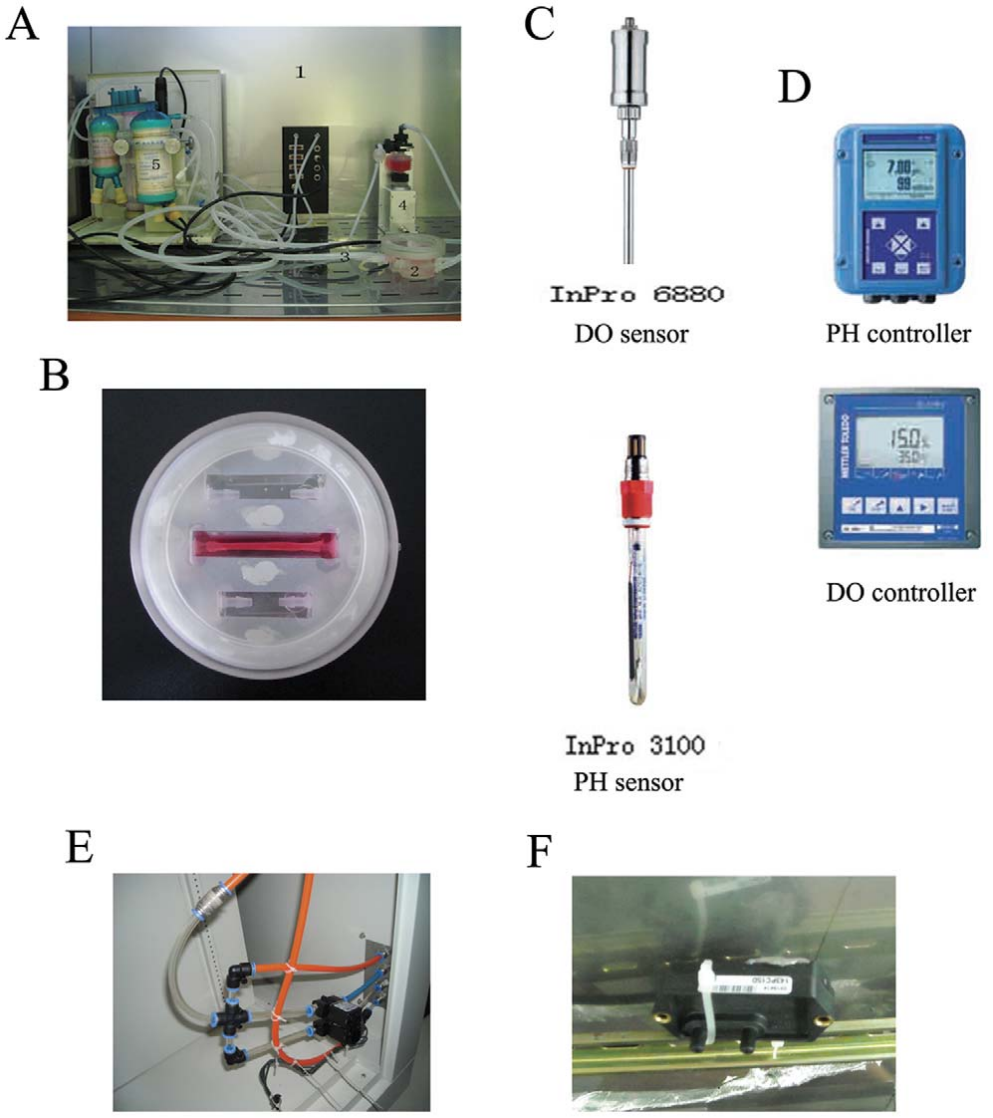
The newly developed integrated vascular bioreactor system. A, (1) the intelligent bionic culture setting; (2) the culture biochamber; (3) silicone tube; (4) the stepper motor-driven pump; (5) the gas/liquid and liquid/liquid exchanger. B, the high-magnification image of the culture chamber. C, the DO sensor and PH sensor. D, the DO controller and PH controller. E, the flow control valve to control the carbon dioxide, nitrogen, and oxygen fluid. F, the force monitor.

## Materials and Methods

Reagents were from Sigma (St. Louis, MO) unless stated otherwise.

**Figure 2:**
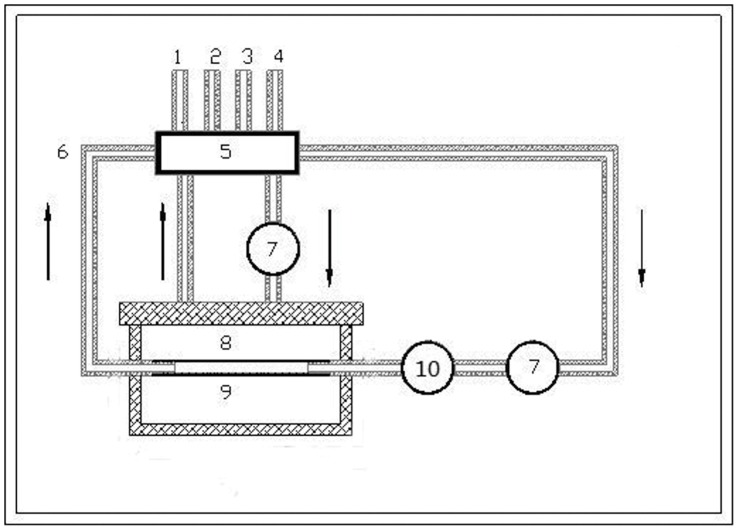
The schema of the perfusion-based bioreactor systems. (1) air inlet; (2) air outlet; (3) fluid inlet; (4) fluid outlet; (5) the gas/liquid and liquid/liquid exchanger; (6) silicone tube; (7) the stepper motor-driven pump; (8) the culture biochamber; (9) the tissue-engineered vascular graft; Arrow, the liquid flow; (10) the pressure sensor.

### System Setup

The newly developed integrated vascular bioreactor system is shown in Fig. 1. Fig. 2 displays the schema of the perfusion-based bioreactor systems. The major components of the culture system include a culture chamber made of plexiglass, a linear motor-driven pump, and medium circulating system, a control system, and integrated auxiliary devices including a laminar flow hood, a temperature controller, and an ozonizer. All devices are assembled in an enclosure.

**Figure 3:**
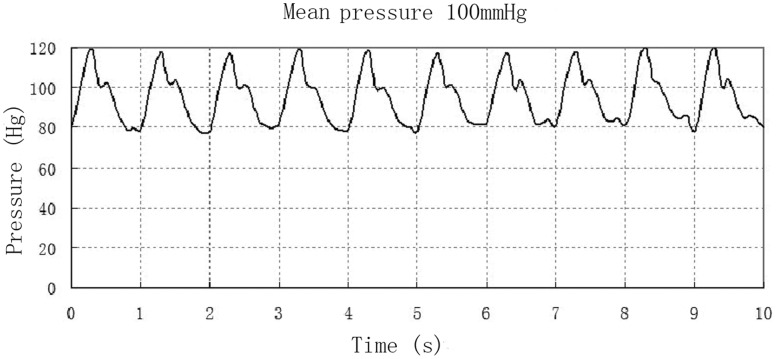
The real-time pressure and pulse rates generated by the motor-driven pump monitored by a baroceptor.

This bioreactor system exerting both compression and medium perfusion is machined from Acrylic organic glass. All screw caps are fitted with a silicone rubber O-ring (Jinan Medical Silicone Rubber; Jinan, China) to seal hermetically. Inlet and outlet ports as well as other fluid ports in this system are standard Luer. Holes of 2 mm in diameter are machined in the upper and basal loading plates for the medium to percolate through.

**Figure 4:**
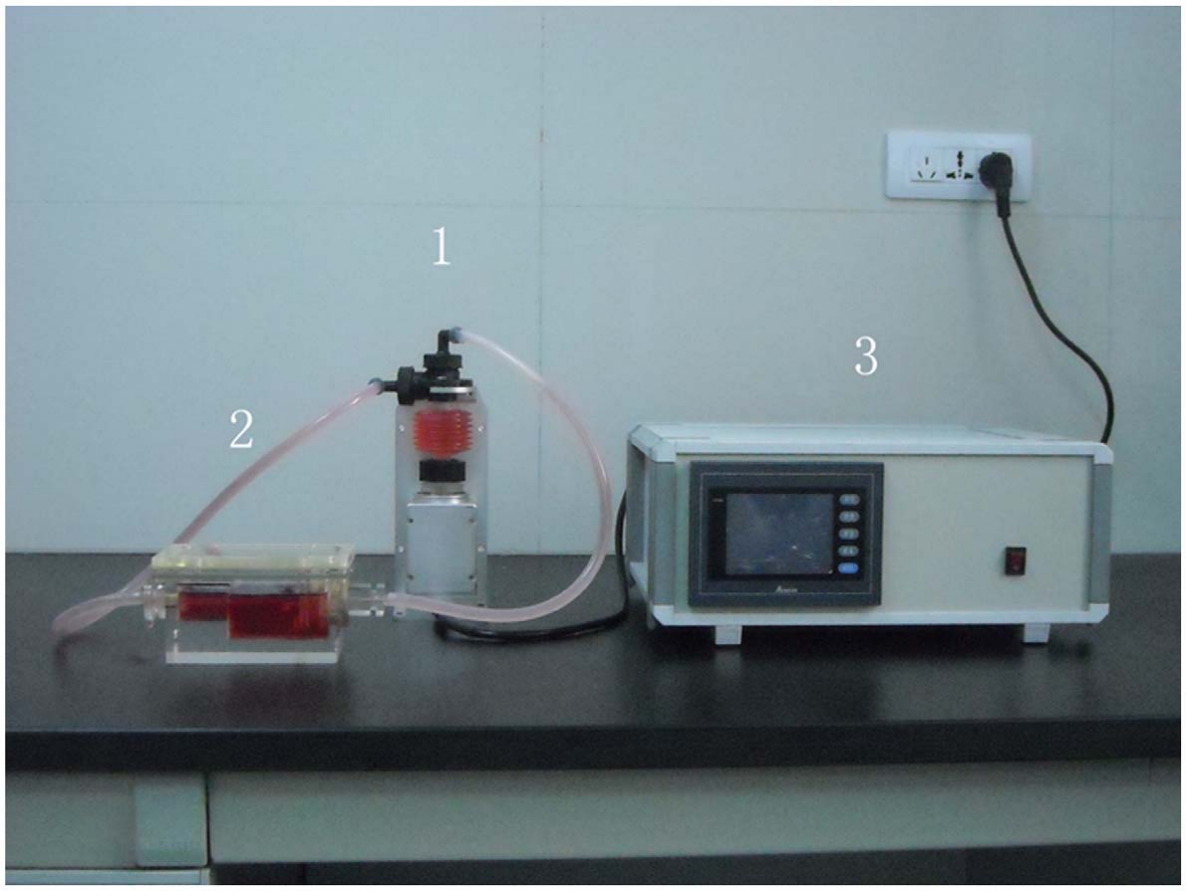
A, The non-computer-controlled culture system: (1) the linear motor-driven pump; (2) the culture chamber; (3) the instrument control system.

The medium circulating system include a medium reservoir, a gas/liquid and liquid/liquid exchanger (Xijing Medical Application Co. LTD, Xian, China), a single-channel pulsatile pump, a multi-channel pulsatile pump (Jiyue Tech; Chongqing, China), a pH sensor (pHG5202, Xuetong Instruments; Guiyang, China), and a dissolved oxygen (DO) sensor (OXY5402, Xuetong Instruments; Guiyang, China). This system consists of two circulating loops: an incubating loop and a medium-replenishing loop. In the incubating loop, a multi-channel pulsatile pump continuously drives the medium along the flow path to supply oxygen and nutrients to the cultures in the bioreactors. In fact, a stainless-steel piston is sealed with silicone-rubber fingercot and then attached to a linear motor-driven pump (45ZWN24-30W/66JB, Haydon Instrument, Changzhou, China) which is integrated with a position detector and a force monitor (S-DEML, Keli Electric, Ningbo, China). The motor-driven pump delivers a peak force of 98 N, a maximum speed of 300 mm/s, and a work travel of 0.1 mm–48 mm. The change of position and force of the system is monitored and transferred to a digital controller as mentioned later. In the medium-replenishing loop, a single-channel pulsatile pump drives the fresh medium into the gas/liquid and liquid/liquid exchanger to replenish the oxygen and nutrients in the incubating loop and removes the waste product. The liquid/liquid exchanger is mainly assembled with two hollow fiber filters sealed in a housing which had room for the medium fluid in the incubating loop. One filter overlaid with narrow stripes is a hydrophilic bio-semipermeable membrane cluster as a nutrient-exchange interface, while the other filter is a hydrophobic bio-semipermeable membrane cluster as a gas-ingredient exchange interface. Fresh medium and sterile gas flow through the inner tubing of the two filters, respectively, through which the nutrition ingredients and required gas ingredients are supplied from fresh medium and gas to the incubating medium; meanwhile, metabolic products are removed. A pH sensor and a DO sensor are sterilized with ethylene oxide, and they access the incubating loop at the medium outlet port. Then the pH and DO concentrations are monitored and transferred to a digital controller for gas-fluid and ingredients controlling. As a result, desirable culture conditions can be maintained in the bioreactors to promote cell culturing.

**Figure 5:**
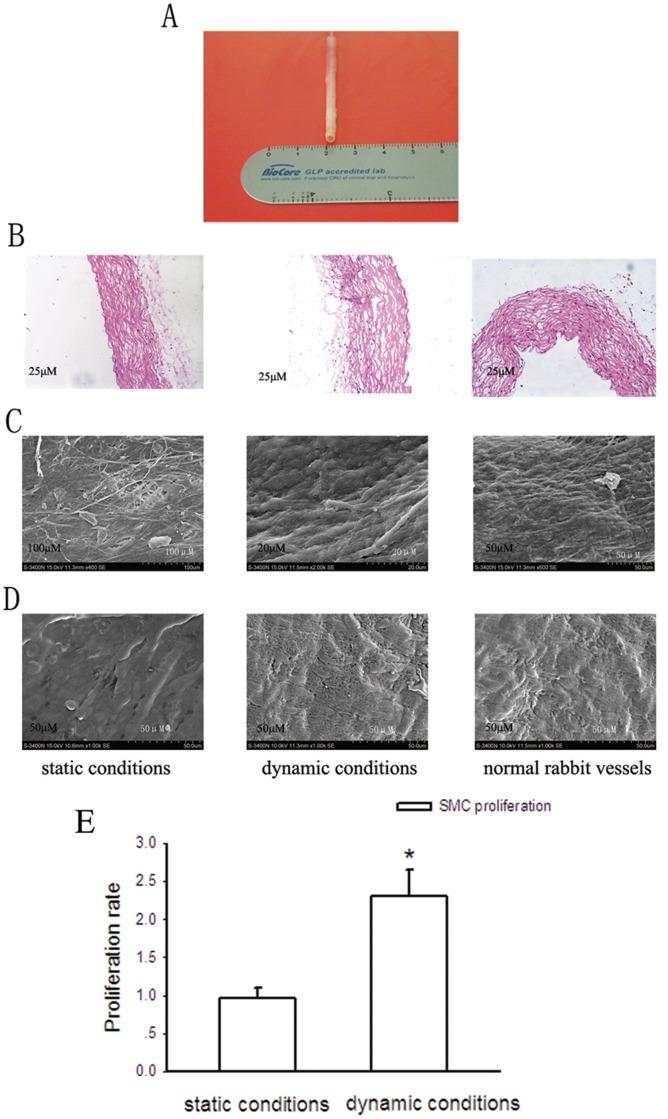
The phenotypes of the newly developed small-diameter vessel cultured for 2 weeks. A, the gross appearance of the newly developed vessel. B, HE staining analysis of histologic sections of the newly developed vessels. C and D, SEM analysis of endothelial cells and smooth muscle cells, respectively. E, the comparison of the survival of SMCs cultured under dynamic conditions with that of SMCs cultured under static conditions determined by MTT assay after 2-week culture. Bars are mean ± S.D. from three independent experiments. *Significantly different from static conditions, *P*<0.05.

The control system is designed to control the medium and gas fluid, the duty cycle of the motor-driven pump, the temperature, and the sterility. The instrument control system, which is based on a ladder-diagram programmed microprocessor control unit (C8501F, Cygnal, USA), drives pulsatile pumps and linear motors with different independent operating parameters; thus, engineered constructs in different bioreactors can be independently subjected to different mechanical stimuli. The pressure control is achieved as the real-time pressure of the medium in the lumen of the constructs is monitored by a pressure sensor (142PC15D, Honeywell, USA). The loading regime in the control system was described as deformation (compression and strain), frequency, and deformation-time waveforms (e.g., sinusoidal wave, triangular wave, square wave, and trapezoidal wave). In order to maintain the cell culture environment, feedback control structures are used to adjust the pH and oxygen content of the medium. The measured pH sensor and dissolved oxygen sensor values are sent to the control system to adjust the electromagnetic flow control valve to control the carbon dioxide, nitrogen, and oxygen fluid. The basic principles are listed as follows:

1. Default pH setting of 7.2–7.4 can be adjusted as needed; when pH<7.2, the nitrogen and oxygen-mixed gas (1∶1) flowed and stopped when pH = 7.3; when pH>7.4, the carbon dioxide flowed and stopped when pH = 7.3.
2. Oxygen content range can be set as needed, e.g., 6.1–7.5 mg/L in following experiments. When oxygen content is lower than 6.1 mg/L, the oxygen flows and stops at the median of the setting (6.8 mg/L); when oxygen content is higher than 7.5 mg/L, the nitrogen flows and stops when oxygen content is 6.8 mg/L. Auxiliary equipments to the system include a laminar flow hood, a temperature controller, and an ozonizer, all of which are integrated in the enclosure, maintaining the chamber at constant temperature and cleanliness via the control system.

**Figure 6:**
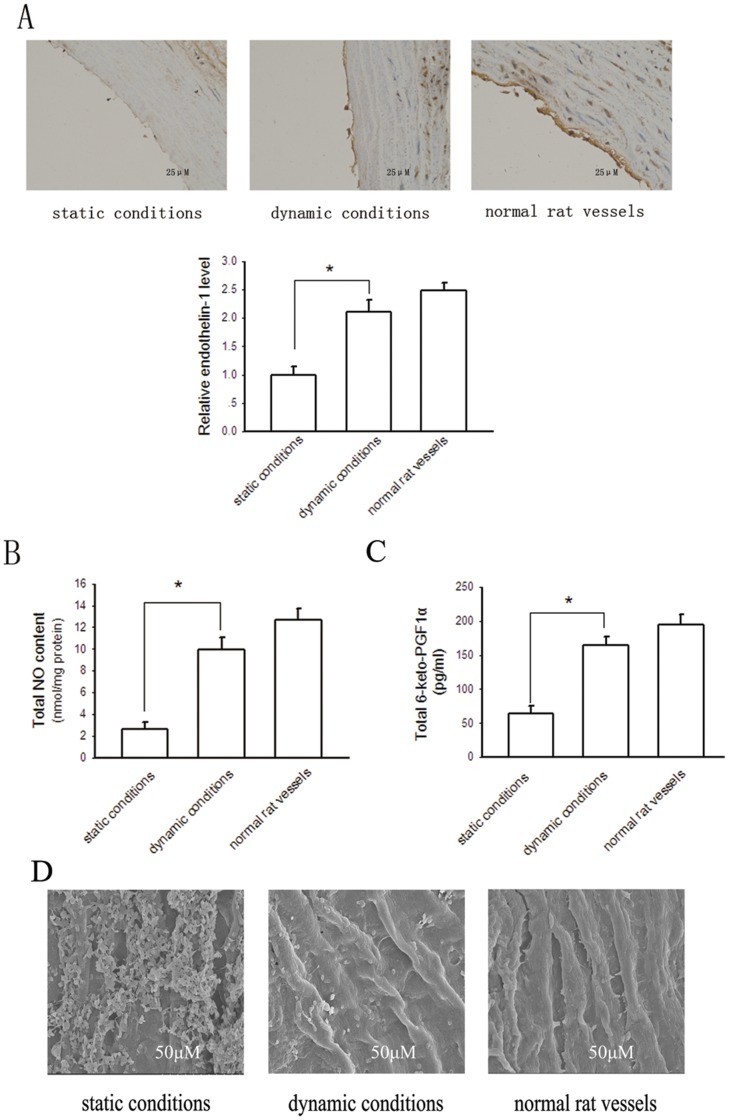
Determination of endothelin-1, NO and PGI2 secretion of vessels. A, immunohistochemistry for endothelin-1 expression of the newly developed small-diameter vessel. Bars are mean ± S.D. from three independent experiments. *Significantly different from static conditions, *P*<0.05. B, total NO secretion determined by use of a chemiluminescence NO detector. Bars are mean ± S.D. from six independent experiments. *P<0.05. C, total PGI2 secretion determined as its metabolite 6-keto-PGF_1α_ assayed by ELISA. Bars are mean ± S.D. from three independent experiments. *P<0.05. D, the morphology of platelet adhesion on the inner lumen surface of tissue engineered vessels was examined by SEM.

### Functional Analysis

The novel perfusion-based bioreactor can impart mechanical stimuli similar to that present in native arteries to vascular constructs. The pressure control is achieved by computer controller-controlled the linear motor-driven pump. The real-time pressure of the medium in the lumen of the constructs is monitored by a pressure sensor (CM53004AS, Zhongwei Tech; Hongkong, China). Performance test showed that pulsatile perfusion actuated by the motor-driven pump was stable. The stretching frequency was 0∼4 Hz. The maximum delivery capacity was approximate 10 mL for one stretching, which could meet the requirements of culture medium cycling in the vascular tissue engineering. As shown in Fig. 3, this computer-controlled bioreactor was able to generate pulsatile flow rates similar to physiological conditions. The real-time pressure monitored by the pressure sensor showed that this bioreactor system could generate physiological pressure, as the systolic pressure was recorded at 120 mmHg, the diastolic pressure was 80 mmHg, and the pulsatile rate was retained at 60 times per minute.

**Figure 7:**
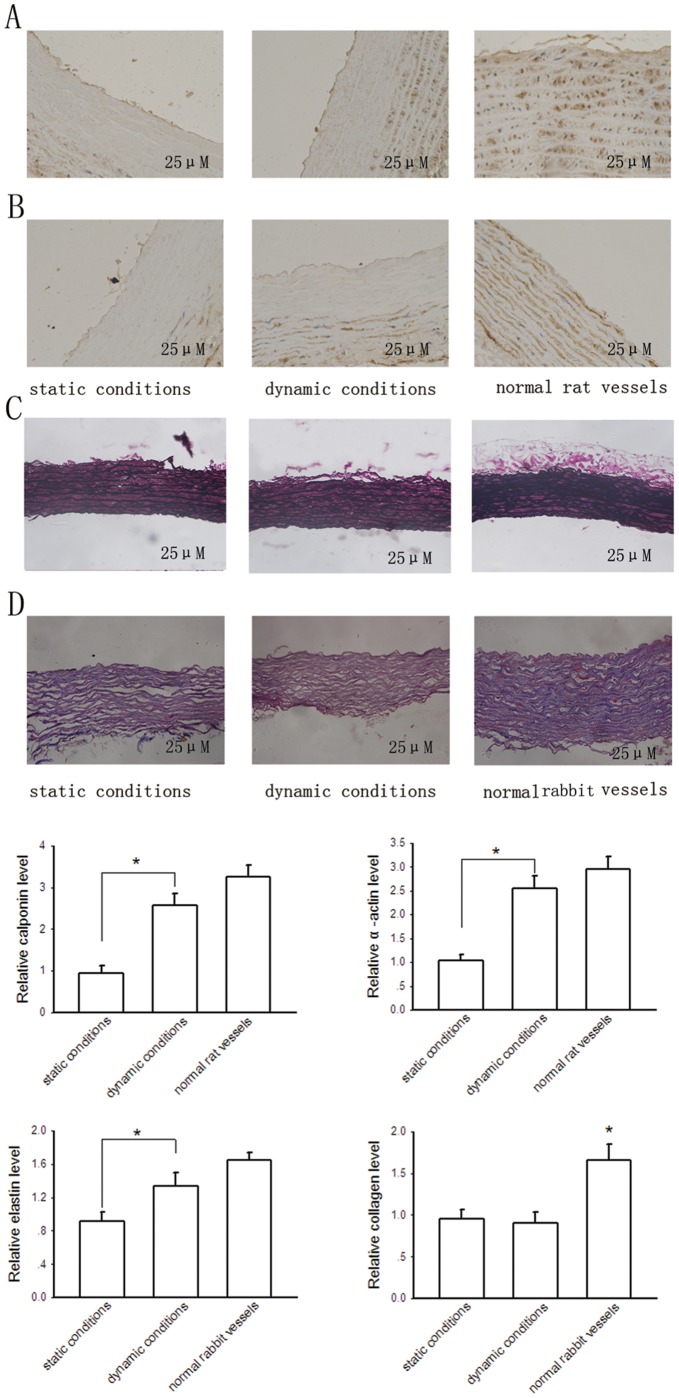
Biochemical assay of newly developed grafts. A and B immunohistochemistry for calponin and smooth muscle α-actin, respectively. C, elastica van Gieson staining of elastin. D, determination of collagen deposition of newly developed grafts by Masson trichrome staining. Bars are mean ± S.D. from three independent experiments. *P<0.05.

### System Setup of the Non-computer-controlled System

The non-computer-controlled culture system mainly consists of a culture chamber made of plexiglass, a linear motor-driven pump and the instrument control system (Fig. 4). This culture system exerting both compression and medium perfusion is machined from Acrylic organic glass. All screw caps are fitted with a silicone rubber O-ring (Jinan Medical Silicone Rubber; Jinan, China) to seal hermetically. Holes of 2 mm in diameter are machined in the upper and basal loading plates for the medium to percolate through. A stainless-steel piston is sealed with silicone-rubber fingercot and then attached to a linear motor-driven pump (45ZWN24-30W/66JB, Haydon Instrument, Changzhou, China). The motor-driven pump delivers a peak force of 98 N, a maximum speed of 300 mm/s, and a work travel of 0.1 mm–48 mm. The culture chamber, the motor-driven pump and the instrument control system were placed in an incubator. The instrument control system, based on a ladder-diagram programmed microprocessor control unit (C8501F, Cygnal, USA), drives pulsatile pumps and linear motors with different independent operating parameters. Therefore, vessels can be independently subjected to different mechanical stimuli.

**Figure 8:**
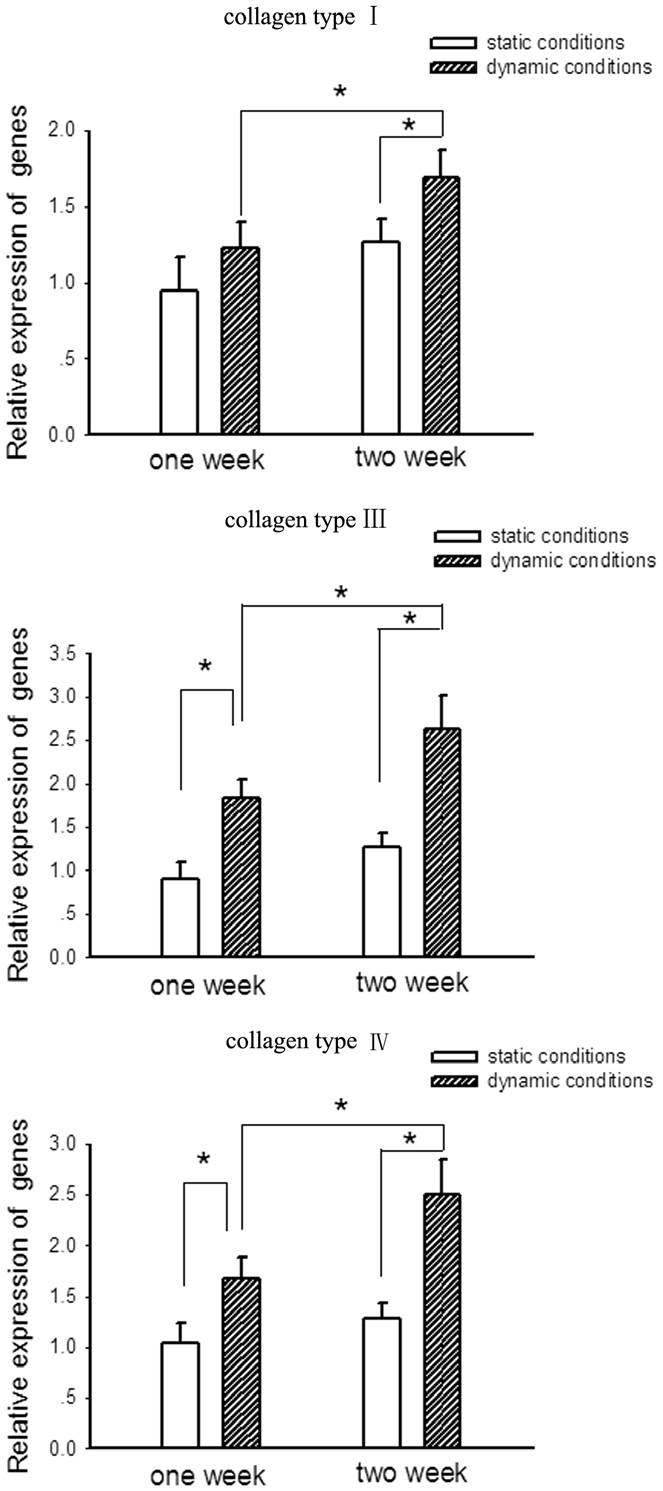
qRT-PCR analysis of collagen-related gene (Collagen type I, III and IV) expression in cells cultured static conditions and dynamic conditions at indicated times. Bars are mean ± S.D. from three independent experiments. *P<0.05.

### Scaffold Preparation

Aortas from rabbits were immersed in 4°C sterile PBS (Gibco, USA) for 2 hrs. The vascular adventitia was stripped under sterile conditions. The blood vessels with complete wall, the length of 5 cm and the diameter approximately at 4 mm were isolated, washed with 4°C sterile PBS solution for 5 times, and then placed into a sterile centrifuge tube of 50 mL. PBS solution of 40 mL containing 2.5 mL/L TirtonX-100 (Sigma, USA), 2.5 g/L sodium deoxycholate (Amresco, USA), 0.2 g/L EDTA (Gibco, USA), 0.1 g/L RNase (Sigma, USA) and 0.2 g/L deoxyribonuclease (Sigma, USA) was added into the centrifuge tube, followed by sustained oscillation in a constant temperature shaker (200 r/min) at 37°C. The mixed liquid was replaced every 24 hrs. After 48 hrs of period, the obtained acellular aortic scaffolds were washed with PBS for three times and stored at 4°C under sterile conditions.

**Figure 9:**
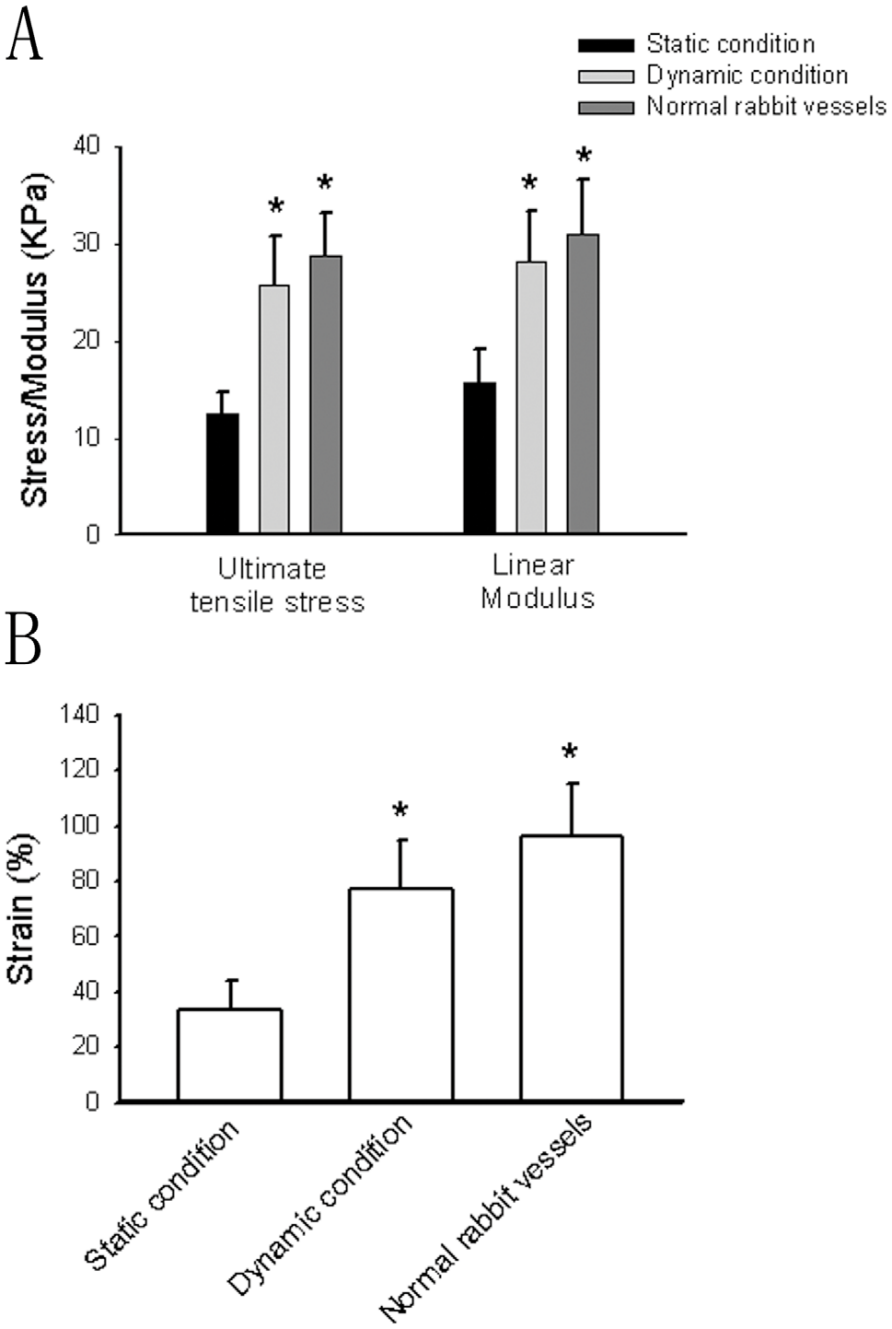
The mechanical properties of the constructs and normal rat vessels as measured using biaxial tensile testing of ring sections of the constructs. Bars are mean ± S.D. from four-seven independent experiments. *Significantly different from static condition group, *P*<0.05.

### Primary Cell Isolation, Culture, and Seeding

The isolated culture and passage of rat aortic SMCs and ECs refers to previous reports [9], [10], respectively. Rat aortic SMCs or ECs of 2nd–5th generation were collected and 400 µL of DMEM culture medium (Gibco, USA) with 10% fetal calf serum (FCS) (Gibco, USA) was add to prepare 6×10^6^/mL cell suspension. The suspension of SMCs was seeded on the external surface of the prepared scaffold and was placed into a incubator (37°C, 5% CO_2_). After 24 hr-incubation period, SMCs were adhered to the external surface of the scaffold. Then, the suspension of ECs was seeded in the lumen of the scaffold uniformly. After ECs were adhered to the lumen completely, the scaffold with seeded cells was placed into the culture chamber of our bioreactor. The culture medium in the outer area of the vessels was DMEM/F-12 supplemented with 10% fetal bovine serum (Gibco) and penicillin 100 (U/ml)/streptomycin (100 µg/ml). The culture medium for culturing SMCs was refleshed twice a week. The medium in the medium reservoir that flew through the inner lumen of the vessels was DMEM supplemented with 10% fetal bovine serum (Gibco) and penicillin 100 (U/ml)/streptomycin (100 µg/ml), which was also refleshed twice a week.

### Scanning Electron Microscopy (SEM)

The developed vascular constructs were fixed overnight with 4% glutaraldehyde and then dehydrated in a graded ethanol series. The grafts were dried, coated with gold and examined by a scanning electron microscope (S3400N, Hitachi Ltd, Japan) operated at 15 kV used to obtain images of these samples.

**Figure 10:**
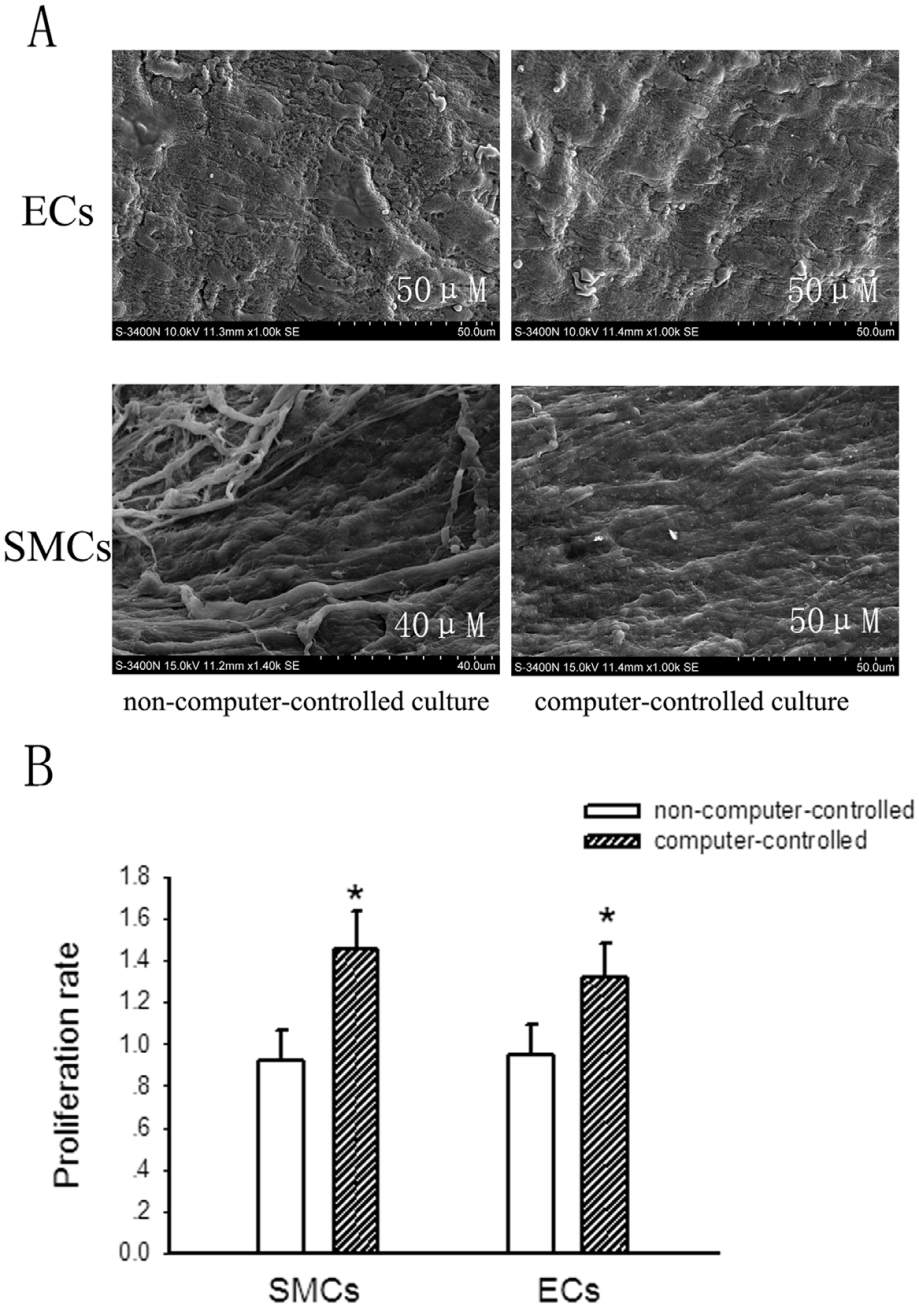
The comparison of cell survival and morphous of constructs between cultured in the computer-controlled bioreactor and the non-computer-controlled bioreactor. A, SEM analysis of endothelial cells and smooth muscle cells cultured both in the computer-controlled and non-computer-controlled system after 2 weeks, respectively. B, the proliferation of endothelial cells and smooth muscle cells cultured both in the computer-controlled and non-computer-controlled system after 2 weeks determined by MTT assay. Bars are mean ± S.D. from three independent experiments. *P<0.05.

### MTT Assay

After 2-week culture, SMCs were isolated from scaffolds referring strictly to a previous study [10]. Cell suspension of 5×10^4^ cells/mL was added into 96-well plates. Cell growth was determined by the MTT quantitative colorimetric assay, as previously reported [11]. OD values were detected by an enzyme-labeled meter.

### Histological Preparation and Analysis

The newly created tissue-engineered blood vessels were fixed in 10% formalin, paraffinembedded, cut in 6-µM-thick cross-sections. Cells and collagen were stained with hematoxylin-eosin (HE) and Masson’s trichrome using Masson trichrome staining Kit (Maxim-Bio, Fuzhou, China) according to the manufacturer’s instruction, respectively. Smooth muscle α-actin, calponin, and endothelin-1 were deparaffinized and immunostained with mouse smooth muscle α-actin (diluted 1∶800), mouse calponin (diluted 1∶100), and endothelin-1 (1∶1000), respectively, then were probed with the secondary antimouse IgG antibody (diluted 1∶500) and visualized with the alkaline phosphatase atandard ABC kit. For determination of elastin, sectioned tissues were stained with elastica van Gieson. All the samples were observed under an inverted microscope and analyzed statistically using IPP (Image-Pro Plus).

### RNA Purification and qPCR

RNA extraction from explanted scaffolds was performed using an RNeasy Mini Kit (QIAGEN VWR, Stockholm, Sweden) following the manufacturer’s instructions. The reverse transcription protocol was restrictly according to a recent study [12]. The analyzed genes included type I collagen (Col1a2, Mm00483888_m1), type III collagen (Col3a1, Mm01254458_g1), type IV collagen (Col4a3, Mm01269207_m1). The highly conserved and universally expressed small nuclear RNA U6 and β-actin genes were used as endogenous qRT-PCR controls.

### Determination of Total Nitric Oxide (NO)

Vascular tissues of normal rat arteries or developed under both static and dynamic conditions were rinsed and homogenized. The tissue NO and its metabolic products (NO_2_ and NO_3_) in the supernatant, were collectively determined by use of a chemiluminescence NO detector (Siever 280i NO Analyzer) as described previously [13].

### Determination of PGI2 Metabolite 6-keto-PGF_1α_

Vascular tissues of normal rat arteries or developed under both static and dynamic conditions were rinsed and homogenized. The level of PGI2 was determinated by ELISA analysis of the level of PGI2 metabolite 6-keto-PGF_1α_ (Cayman Chemicals, USA) by ELISA in a microtiter plate at 25°C using a microplate reader (Dynex Tech., USA) according to the manufacturer’s instruction. Absorbance at 405 nm was detected.

### Platelet Adhesion Assay

The platelet adhesion assay was performed according to a recent report [14] with some modifications. Rabbit whole blood from a healthy rabbit was mixed with 3.8% anticoagulant acid-citrated (1∶1). The mixture was then centrifuged at 1500 rpm for 15 mins to prepare rabbit platelet-rich plasma (rPRP). rPRP of 4 ml was added to a well of a 6-well tissue culture plate. Then the native vessels and newly created constructs cultured under both static and dynamic conditions were immersed into each well with 4 ml rPRP. After 1 h-incubation at 37°C, all samples were dipped in 2.5% glutaraldehyde buffer solution overnight, and then were dipped in PBS 3 times for 15 mins. Then each sample was dehydrated in increasing concentrations of ethanol (30, 50, 70, 90, 95 and 100%) for 15 mins. The morphology of platelet adhesion on the inner lumen surface of each sample was examined by a scanning electron microscope (S3400N, Hitachi Ltd, Japan) operated at 15 kV.

### Mechanical Testing

The constructs were sectioned into 4 mm long segments for mechanical testing immediately after removal from the bioreactor. Vessels cultured under static conditions and normal rabbit vessels were also tested. The mechanical properties were assayed by testing the failure of segments using an Instron 5842 uni-axial mechanical tester equipped with a 5.0 N load cell. Video capture of mechanical testing was collated with force-extension data and segment dimensions to calculate ultimate tensile stress, strain at failure, and linear elastic modulus. Instrumental control and data collection were performed using LabVIEW (National Instruments).

### Statistics

The results were expressed by means ± S.D. Multi-factor analysis of variance was applied using SPSS10.0 software. The difference was statistically significant when *P*<0.05.

## Results

### The General Morphous of the Newly Created Graft and Morphological Analysis

As shown in Fig. 5A, the lumen diameter of our bioreactor-produced vascular vessel was shorter than 4 mm, with the length about 5 cm. The vessel was high patent and the color was comparable to native vessels. As shown by HE staining (Fig. 5B), after 2-week culture, vascular grafts cultured in the dynamic bioreactor system showed normal appearing smooth muscle cells with circumferential orientation in the external areas of the graft and the endothelial layer appeared healthy and nearly confluent. Vascular tissues cultured in the dynamic bioreactor system showed more cell density of both aortic smooth muscle cells and endothelial cells than that of cells cultured under static conditions.

SEM surface analysis showed that after 2-week culture, a complete monolayer of aortic endothelial cells had formed and the cells were more widely distributed than those cultured under static conditions (Fig. 5C). The smooth muscle cells on the external surface of the scaffold extended many projections toward their adjacent cells cultured under dynamic conditions (Fig. 5D). On the contrary, when cultured under static conditions for the same 2–week period, smooth muscle cells formed a spindle shape along the scaffold, and the cell density was very low. Furthermore, Fig. 4E showed that after 2-week culture, the survival of SMCs in the computer-controlled bioreactor was approximately 2.2 folds more than that of SMCs cultured under static conditions. This result suggests that the computer-controlled bioreactor is superior for cultured SMC proliferation compared with the static culture system.

### The Biochemical Phenotypes of the Dynamic Bioreactor System-produced Vessels

Functional ECs can secrete a variety of endothelium-derived relaxing and contracting factors, such as NO, PGI2 and endothlin-1 [15]. Fig. 6A showed that immunohistochemical staining for endothelin-1 was positive near the lumen in the construct. The expression level of endothlin-1 in the graft cultured under dynamic conditions, obviously more than that in the construct developed under static conditions, was comparable to that in native vessels. Fig. 6B and C indicated that the endothelium of the graft developed in our bioreactor could produce considerable NO and PGI2, respectively. The levels of produced NO and PGI2 in the newly created construct were more comparable to those in native vessels than that in tissues cultured under static conditions. These results demonstrate that the endothelium of the vessel developed in our bioreactor system has favourable physiological function.

To evaluate the anti-thrombotic property of the newly developed small-diameter vessels, we performed platelet adhesion assay as the platelet adhesion property is an indicator of the anti-thrombogenic potential of vascular vessels. Fig. 6D showed that a large number of platelets adhered to the endothelial layer of the vascular construct cultured under static conditions, whereas much fewer platelets adhered to the inner lumen of the vessel cultured under dynamic conditions. However, though not significantly, the platelets adhered to the endothelial layer of the vessel cultured under dynamic conditions seemed little more than the normal rat vessel. This result suggests that small-diameter constructs cultured in our bioreactor system have favourable anti-thrombotic property. Nevertheless, as the best culture conditions remain largely unclear, the anti-thrombotic property of our engineered vessels is not completely comparable to that of native vessels.

Calponin and α-actin are two standard markers used to identify cells expressing a SMC phenotype [16]. As shown in Fig. 7, immunohistochemical staining showed that SMCs close to the free surfaces stained positively for calponin (Fig. 7A) and α-actin (Fig. 7B). Pulsatile flow stimuli improved calponin and α-actin production, with calponin and α-actin levels in the dynamic constructs comparing favorably to those of native arteries. These data suggest that SMCs cultured in our bioreactor for 2 weeks are well differentiated and express a contractile phenotype.

The extracellular matrix (ECM) is formed by collagen and elastin mainly produced by SMCs. As shown in Fig. 7C, elastin staining was relatively lower detectable in vascular grafts cultured under static conditions, whereas were both abundant in the external surface areas of the cross-sections of constructs cultured under dynamic conditions and native vessels. Fig. 7D showed the amount of collagen was not significantly different in constructed vessels between those cultured under static conditions and dynamic conditions. This may be due to minimal production of collagen by cells during the short culturing period, and the production of collagen is relatively very low compared with the basal level of collagen in cell-free vascular scaffolds. Furthermore, the amount of collagen in constructed vessels was significantly lower than that in native vessels, possible due to some loss during the decellularization process of scaffold preparation.

To confirm that collagen is expressed in cultured cells, we sought out to determine the changes in gene expression of the collagen subtypes (Collagen type I, III and IV) according to a recent study [12] and made comparison of the expression level of these genes in cells between cultured under static conditions and dynamic conditions.

Fig. 8 illustrated changes in gene expression of the collagen subtypes over the period of development of small diameter vessels. The results indicated the expression levels of collagen I, III and IV were all upregulated in cells cultured under both static conditions and dynamic conditions at 2 week. However, static conditions imposed a relatively minimal stimulation of these genes expression. At 1 week, the levels of gene expression for both collagen III and collagen IV in cells cultured under dynamic conditions were greater than those cultured under static conditions, and were even greater at 2 week, while there was no significant difference in collagen I expression in cells between cultured under static conditions and dynamic conditions at 1 week. These results suggest that this dynamic bioreactor is superior for collagen-related genes expression.

### Mechanical Properties

After 4-week culture, the mechanical properties of engineered constructs were tested. As shown in Fig. 9A and Fig. 9B, the ultimate tensile stress, linear modulus, strain at failure of the constructs cultured under dynamic stations and normal rabbit vessels were significantly higher than those cultured under static conditions. The mechanical properties between the constructs cultured under dynamic stations and normal rat vessels had no statistic differences. These results suggest that the mechanical properties of constructs cultured by the present bioreactor are similar to those of normal vessels.

### The Culture Conditions of the Present Computer-controlled Bioreactor is Superior to the Traditional Non-computer-controlled Perfusion Bioreactor

After 2-week culture, the morphous of constructs cultured in the computer-controlled bioreactor and the non-computer-controlled bioreactor were analyzed by SEM. Generally, there had no significant differences in the morphous between constructs cultured in the computer-controlled bioreactor and those cultured in the non-computer-controlled bioreactor after a short-culture period (Fig. 10A). To make comparison of cell survival in the bioreactor between with and without computer-controlled, we used MTT assay to determine cell survival after 2-week culture. The procedure of isolated culture of smooth muscle cells and endothelial cells refers strictly to reported studies [9], [10]. Fig. 10B showed that after 2-week culture, the survival of SMCs and ECs in the computer-controlled bioreactor was approximately 1.4 and 1.3 folds more than that of SMCs and ECs in the non-computer-controlled bioreactor, respectively. This data suggests that the computer-controlled bioreactor is superior for cultured cell proliferation compared with the non-computer-controlled bioreactor.

## Discussion

In this study, we successfully develop small-diameter vascular constructs using our integrally designed computer-controlled bioreactor system. This computer-controlled bioreactor system can impart physiological mechanical stimuli and fluid flow similar to that present in native arteries to the cultured grafts. Results from biochemical and mechanical assay confirm the feasibility of the newly developed bioreactor system for tissue engineering of small-diameter vascular vessels.

Various previously reported devices of vascular tissue engineering apply a mechanical ventilator or a peristaltic pump to enable the formation of variable pulsatile waveforms [17], [18]. The present device, however, applied a motor-driven pump to enable the stable pulsatile perfusion. The computer-controlled motor-driven pump used in our system is superior to other pumps and ventilators as it can be capable of achieving a bionic pulsating effect of intermittent medium flow other than maintaining a certain fluid pressure. Furthermore, the present perfusion system can effectively export up to 10 mL medium for one stretching of the pump and mimick the mechanical stimuli according to physiological needs.

Small-diameter vessels are pressurized to physiological mean pressure around 60 mmHg [19], [20]. Actually, we used pressures reported in the present study as mechanical stimulation according to most other studies [21], [22]. However, this does not mean that exposure of 120 mmHg of systolic and 80 mmHg of diastolic pressure is the favorable mechanical stimuli for development of tissue-engineered small-diameter vascular constructs. Many studies have detected the effects of various culture conditions on tissue-engineered vascular grafts, however, the optimal stimuli remain still elusive. Many studies have detected the effects of various culture conditions on tissue-engineered vascular grafts, however, the optimal stimuli remain still elusive. In the early stages of development of vessels, stimuli with low pressure and high-frequency pulsatile flow contribute critically to the differentiation and development of tissue-engineered cardiovascular tissues [23]. Thus, increasing pressure level (up to the physiological range) submitted to tissue-engineered grafts is of great importance for maturation of vascular tissues. Our computer-controlled bioreactor system can allow adjustment over a varying range of pressures and pulse rates, thus making great improvements in tissue engineering of vascular vessels. Therefore, this new bioreactor system is suitable for evaluating a favorable condition for development of tissue-engineered vascular constructs. Nevertheless, future studies are in urgent need to explore the best mechanical stimuli for tissue engineering of small-diameter vascular constructs.

In this study, the highlight of the bioreactor system is the automatic feedback controlled substance-exchange system, which consists of real-time medium sensors, an intelligent controller, a gas magnetic valve, and a gas/liquid and liquid/liquid exchanger. At the core of this system, the substance exchanger integrates the principles of both a hemodialyzer and a lung machine. Briefly, on the basis of the controlling principles, sterile O_2_ and CO_2_ are perfused into the exchanger according to DO and pH sensors, keeping the pH and oxygen content steady at predetermined scales. As the media are continuously perfused in both the fresh medium loop and the incubating medium loop, nutrients and wastes are diffused through the bio-semipermeable membrane along with the concentration gradient. Meanwhile, the bio-semipermeable membrane allows molecules below 14000 Da to pass freely. Therefore, this feedback-controlled substance-exchange system helps maintain the biochemical environment within a determined scale. Specifically, this design of automatic nutrient-supplying means more strict hermetic sealing during a long-term culture.

A number of studies including recent ones have demonstrated pulsatile bioreactor culture systems for engineered small diameter vessels [4], [24]. However, the present computer-controlled perfusion system is superior to other pulsatile bioreactors due to its precise regulation of mechanical and chemical stimulation imparted to engineered small diameter vessels. The instrument control system drives pulsatile pumps and linear motors with different independent operating parameters, thus engineered constructs can be independently subjected to different mechanical stimuli. The pressure control is achieved as the real-time pressure of the medium in the lumen of the constructs is monitored by a pressure sensor. In this study, the highlight of the bioreactor system is the automatic feedback controlled substance-exchange system, which consists of real-time medium sensors, an intelligent controller, a gas magnetic valve, and a gas/liquid and liquid/liquid exchanger. At the core of this system, the substance exchanger integrates the principles of both a hemodialyzer and a lung machine. Briefly, on the basis of the controlling principles, sterile O_2_ and CO_2_ are perfused into the exchanger according to DO and pH sensors, keeping the pH and oxygen content steady at predetermined scales. As the media are continuously perfused in both the fresh medium loop and the incubating medium loop, nutrients and wastes are diffused through the bio-semipermeable membrane along with the concentration gradient. Meanwhile, the bio-semipermeable membrane allows molecules below 14000 Da to pass freely. Therefore, this feedback-controlled substance-exchange system helps maintain the biochemical environment within a determined scale. Specifically, this design of automatic nutrient-supplying means more strict hermetic sealing during a long-term culture.

One challenge in development of vascular tissue-engineered bioreactors is that they must effectively transfer a homogeneous mix of nutrients, waste metabolites and gases to the grafts to promote cell growth and maturation [18]. Another fundamental technical requirement is that handling of these bioprocesses should be simple to minimize the potential for contamination throughout culture period. However, most previously reported perfusion-based bioreactors are fitted in an incubator with a separate oxygenator to supply the transfer of mass oxygen [17], [25], [26], [27], thus complicating the replacement of culture medium and increasing the possibility of contamination. In the present study, our medium circulating system eliminates the complicated replacement of culture medium in traditional vascular tissue engineering, thus reducing the possibility of contamination as well as improving the automation of tissue culture. Specifically, this integrally designed computer-controlled bioreactor system without the accessory of an additional incubator is easily industrially manufactured and has the potential in large-scale production of vascular grafts.

Tissue engineered small-diameter vessels with high patency and antithrombotic properties required is really a great challenge. In the present study, our bioreactor successfully developed high patent vascular grafts with the diameter lower than 4 mm. The results from immunohistochemistry, enzymatic assay of nitrate reductase and ELISA showed that ECs in the developed vessels could secrete large amounts of endothelin-1, NO and PGI2, respectively. These results suggest that our bioreactor can impart physiological stimuli that effectively promote the differentiation and maturation of ECs. Furthermore, the level of NO and PGI2 secretion was significantly more than that in vessels cultured under static conditions. NO or PGI2 released by the vascular endothelium has been widely accepted to be responsible for the vascular relaxant properties [28], [29]. Thus, the high NO and PGI2 production of the newly developed vascular grafts similar to the physiological vessels may account for the achievement of the high patent feature of our tissue engineered small-diameter vascular constructs. A leak-proof confluent ECs monolayer (endothelium) has been confirmed to be a prerequisite for a vascular graft to resist thrombosis *in vivo*. Thus, the development of a functional integrity of the endothelium in the construct lumen is a critical challenge for tissue engineering of small-diameter vascular grafts for long-term patency. In this study, dynamic culture of ECs appeared confluent in the lumen of the newly developed vessels, contributing to the vessel antithrombotic function. Besides, these functional ECs could produce mass NO and PGI2, both of which have the potential in anti-thrombosis due to inhibition of platelet adhesion to the vascular endothelium [30]. Platelet adhesion study confirmed that small-diameter constructs cultured in our bioreactor system have favourable anti-thrombotic property. Taken together, our bioreactor system can successfully develop functional small-diameter vascular grafts with high patent character and potentially antithrombotic properties.

A functional vascular graft must also possess mechanical properties such as both compliance and burst pressure similar to the native vessels in order to sustain anastomotic pressure without rupture. The mechanical properties of a functional vessel are mainly derived from the composition and orientation of ECM, consisting mainly of intermixed collagen and elastin mainly produced by vascular SMCs. The collagen fibers are the main components providing the tensile stiffness of a vessel, and elastin is responsible for the elastic properties of a blood vessel. However, there still remains a great challenge in the *in vitro* production of elastin fibers [31]. In this study, the SMCs under the culture condition of our bioreactor stained positively for α-actin and calponin, indicating that these cells were well differentiated and expressed a contractile phenotype. The two most abundant proteins in a native blood vessel are collagen and elastin, which are mainly produced by SMCs. Together with SMCs, they confer to vascular wall strength, elasticity and ability to retain its shape [32]. In the present study, Masson staining assay and immunohistochemical analyses showed that the elastin and collagen deposition in the newly developed graft produced by our bioreactor was comparable to native vessels and obviously more than that cultured under static conditions. Furthermore, the mechanical assay of the newly developed constructs confirms the effectiveness of the present bioreactor system.

Various human-derived cells instead of vascular cells from rat or rabbit have used as cell sources for engineered small diameter vessels. For example, bone marrow–derived cells [33] and adipose-derived stem cells [24] have been confirmed to have the potential to regenerate vascular tissues and improve patency in tissue-engineered small-diameter vascular grafts. Translating these cells-derived engineered small diameter vessels into humans may overcome tremendous obstacles such species and age differences. However, the present study was designed to develop a novel pulsatile perfusion-based bioreactor for development of engineered small diameter vessels. Our future studies will stress on exploration and confirmation of the best mechanical and chemical condition for tissue engineering of small-diameter vascular grafts. Nevertheless, bone marrow-derived cells and adipose-derived stem cells may be better alternative cell sources for our future *in vivo* studies.

In conclusion, in the present study, we successfully develop an integrally designed vascular bioreactor system to construct functional small-diameter vascular vessels under the precise control of the computer-based manipulation. Biochemical and mechanical assay of newly developed grafts confirm the feasibility of the bioreactor system for vascular engineering. Furthermore, our novel bioreactor system may be a potential alternative for tissue engineering of large-scale small-diameter vascular vessels for clinical use.
